# Quadrupedal locomotor simulation: producing more realistic gaits using dual-objective optimization

**DOI:** 10.1098/rsos.171836

**Published:** 2018-03-07

**Authors:** William Irvin Sellers, Eishi Hirasaki

**Affiliations:** 1 School of Earth and Environmental Sciences, The University of Manchester, Manchester, UK; 2 Primate Research Institute, Kyoto University, Inuyama, Japan

**Keywords:** gait, simulation, lateral stability, optimization

## Abstract

In evolutionary biomechanics it is often considered that gaits should evolve to minimize the energetic cost of travelling a given distance. In gait simulation this goal often leads to convincing gait generation. However, as the musculoskeletal models used get increasingly sophisticated, it becomes apparent that such a single goal can lead to extremely unrealistic gait patterns. In this paper, we explore the effects of requiring adequate lateral stability and show how this increases both energetic cost and the realism of the generated walking gait in a high biofidelity chimpanzee musculoskeletal model. We also explore the effects of changing the footfall sequences in the simulation so it mimics both the diagonal sequence walking gaits that primates typically use and also the lateral sequence walking gaits that are much more widespread among mammals. It is apparent that adding a lateral stability criterion has an important effect on the footfall phase relationship, suggesting that lateral stability may be one of the key drivers behind the observed footfall sequences in quadrupedal gaits. The observation that single optimization goals are no longer adequate for generating gait in current models has important implications for the use of biomimetic virtual robots to predict the locomotor patterns in fossil animals.

## 1. Background

Symmetrical quadrupedal gaits are a fundamental component of the locomotor repertoire of almost all terrestrial quadrupeds. However, while interest in these gaits can be traced back to the origins of dressage in the 16th century, we are still unable to categorically define why a particular animal would choose a particular gait at a particular speed [1]. In the context of evolution, it is easy to see the advantages of choosing gaits that minimize energetic cost and hence reduce the overall energy budget for an animal. Indeed energy efficiency has been found to be the primary driver for broad categories of gait choice (walk, trot, run) in some studies (e.g. [2,3]), although this finding is not universal (e.g. [4,5]). However, there are other factors that are also important for gait choice such as rate of travel, manoeuvrability, various measures of stability, and the size and structure of the body [6]. Quadrupedal footfall sequences have been measured for a large range of animals in at least 143 tetrapod genera [7] but it still remains difficult to distinguish the importance of gait choice criteria. Energetics have been implicated in dog walking [8]; peak limb forces and metabolic factors are found not to be important for gait selection in horses [9]; balance is suggested as an important driver among primates [10] but there is some disagreement [11,12]; and neural control [13], skeletal loading [4] and limb interference avoiding [14] have all been suggested as alternatives. Primates are a particularly interesting case because they have a very unusual diagonal footfall sequence at walking speeds, and generally they transition from walking directly to galloping without an intermediate trotting phase [15]. Additionally, this diagonal gait pattern is preserved in other locomotor modes such as climbing [16] and even in human bipedal locomotion where arm swinging is diagonally out of phase with footfalls [17] and where diagonal gaits predominate in the short post-natal quadrupedal crawling stage [18].

It is important to distinguish the various uses of the term ‘stability' in mechanics. In this paper, we are referring to stability in the orientation axes. In particular, we are interested in lateral stability which is the tendency for a moving body to recover from small roll perturbations and, therefore, move forward with relatively small oscillations about the longitudinal axis. This use of the term stability (along with longitudinal stability which reduces pitch in the lateral axis, and directional stability which reduces yaw in the vertical axis) is commonly encountered in aeronautics [19] but is equally applicable to legged animals. However, there are different uses of the term stability that are commonly encountered in the gait literature. In particular, gaits are often described as either statically or dynamically stable depending on whether or not the centre of mass is always maintained within the support polygon, and it can be shown that only a subset of creeping gaits can be statically stable throughout the gait cycle [20]. From this we can also define the *stability margin* as the shortest distance from the vertical projection of the centre of gravity to the boundary of the support pattern. This value is positive when a gait is statically stable and becomes negative when the gait is dynamically stable, and can be used as a pragmatic metric for estimating the size of external perturbation that a gait can accommodate without losing the property of being statically stable [21]. The advantage of statically stable gaits is that they maintain the animal in an upright position independent of speed and this property allows extremely low speed locomotion to be possible. However, at higher speeds it becomes possible and ultimately necessary to adopt dynamically stable gaits that require forward movement to maintain the upright position of the animal. Stability in dynamic gaits is then often quantified as the ability to remain upright even when the gait is perturbed in some way, particularly in the context of studies of falling in humans [22]. There are then a large range of different measures that have been used to assess this aspect of stability and these have been extensively reviewed elsewhere [22], with as yet no single measure having been adopted as the best way of assessing stability.

Computer simulations of quadrupedal locomotion are an important tool for investigating locomotion but these tend to be rather abstract models designed to answer particular aspects of the mechanics rather than providing a high level of realism (e.g. scaling effects [23], passive dynamics [24] and gait pattern generation [25]). There are many examples of very sophisticated simulations in the robotics and computer graphics fields but cases where the primary aim is to produce a high biofidelity representation in order to understand the biomechanics of animals are much less common. One of the earliest examples is a quadrupedal horse model produced in 1989 validated against motion capture and force-plate data to explore how well such a model could represent the experimental data [26]. Later examples include a model of a hedgehog validated against cinefluorographic data as a tool to aid reconstruction of fossil animals [27]. This idea has been exploited in other taxa with, for example, models of various quadrupedal dinosaurs used to reconstruct their possible gait patterns [28,29]. An early example in primates is a detailed model of a Japanese macaque developed to investigate the effects of locomotor kinematics on energy cost [30,31], and similar models have been produced for chimpanzees to investigate spontaneous footfall sequence generation [32]. These models work extremely well when they can be trained to mimic experimentally derived data (as is generally the case), but when allowed to spontaneously generate gaits (as is necessary when they are used for fossil reconstruction), the gaits produced are often disappointing, particularly when considered in three dimensions (e.g. [29,32]), and there are issues where the simulation generates a range of possible gaits [28] and it would be beneficial to be able to decide on objective criteria such that there is only one gait that is ultimately likely for a particular morphology and desired locomotor speed.

The aim of this paper is thus to investigate the interaction of lateral stability and energetic criteria in an anatomically and physiologically realistic simulation. It is hoped that this will improve the apparent realism of the simulation, and quantify the importance of lateral stability as a factor that controls footfall phase relationships and identify to what extent it might drive gait choice. This requires the use of multi-objective optimization which is an active area of research because simple approaches, where a composite score is assigned as a weighted combination of the scores for each separate objective, are often not particularly effective [33]. We have used such linear combinations of scores to explore the relationship between energy efficiency and forward velocity in bipedal locomotion previously [34], but more recently we have been experimenting with nonlinear approaches such as using hard coded constraints to drive the simulation to explore specific regions of the search space [35], and this latter option is the approach adopted here.

## 2. Material and methods

The chimpanzee musculoskeletal model has been described in detail elsewhere [32]. In brief, this model was created from a full body CT scan of an adult male common chimpanzee, with no obvious musculoskeletal defects, that died of natural causes at the Welsh Mountain Zoo in the UK. The CT scan was used to create a skeletal model and a skin outline. The skeletal model was used to define joint positions, muscle paths and limb contact points. Muscle geometrical parameters were derived from the literature (primarily [36]). Joint excursion limits were set based on those reported for quadrupedal gait [37]. Mass properties were derived geometrically from the skin outline using chimpanzee-specific segment densities [38]. The muscle model used in this paper reflects changes in the simulation software to improve the numerical stability of the muscle contraction force calculation and there have been some small alterations in the muscle paths to more closely reflect the skeletal anatomy, however these changes have very little effect on the performance of the model. The full model specification is in the human readable XML file supplied as the electronic supplementary material.

To test the effect of optimization goal and footfall patterns on the gait produced by the model, we created two sets of starting conditions. The first was based on chimpanzee kinematics recorded previously [37], which provided the starting segment orientations and segment velocities needed for the normal chimpanzee diagonal sequence gaits. We were then able to use our standard genetic algorithm based machine learning algorithm to find activation patterns that could generate continuous diagonal sequence gait based on these starting conditions [32]. A second set of starting conditions was required to produce lateral sequence gaits because none of the video clips we were able to record showed chimpanzees using lateral sequence gaits (although they do occasionally do so [39]). Fortunately, lateral sequence gaits are commonly generated using our *de novo* gait generation procedure [32], and a set of segment orientations and segment velocities appropriate for lateral sequence gaits could be extracted from a previous simulation and used to generate lateral sequence gait using the new model. We then applied our standard gait morphing methodology [40] to investigate the effect of different optimization criteria on the different footfall patterns. The primary driver was energetic efficiency implemented as maximizing the distance travelled forward for a specified metabolic energy limit. A laterally stable model is not allowed a large degree of lateral movement so lateral stability as an optimization goal was implemented as a hard limit on the maximum lateral velocity that the model was allowed to generate. The models were also speed limited to a Froude number (speed^2^/(g × hip height)) of 0.25 because this is just over the minimum cost of transport speed (0.23) previously found [41] and in the middle of the range (0.2–0.3) chosen in experimental studies [42].

**Figure 1.**
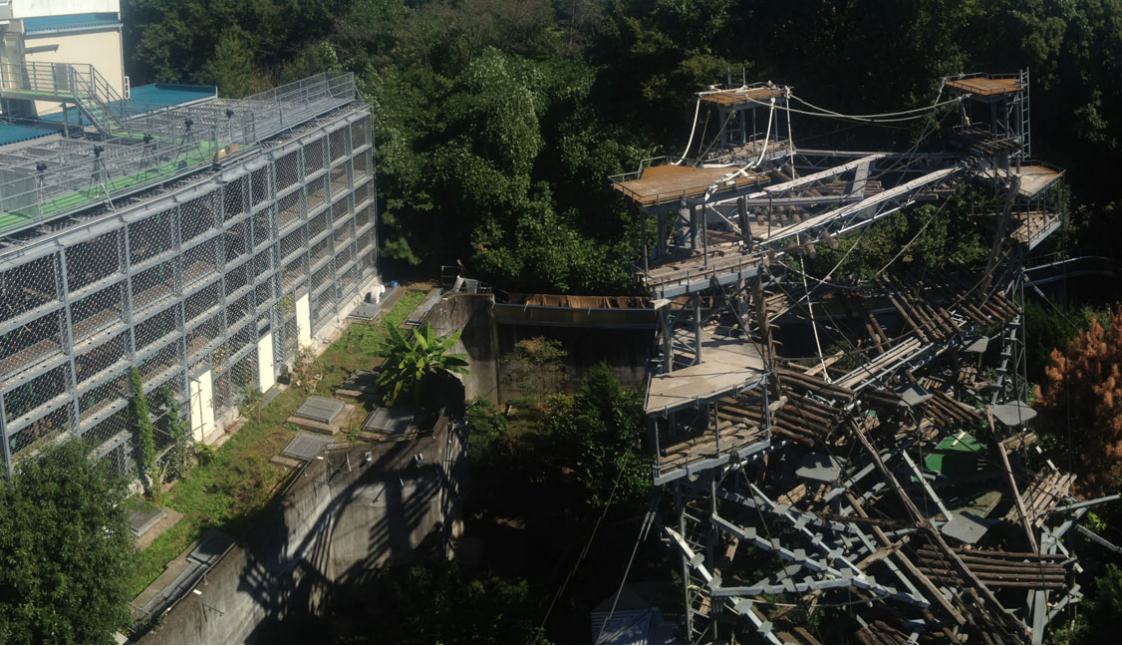
Camera set-up used to film the chimpanzees in their enclosure at the Kyoto University Primate Research Institute. The horizontal platforms used by the chimpanzees are the horizontal planks running between the three towers forming a triangle at each level.

**Figure 2.**
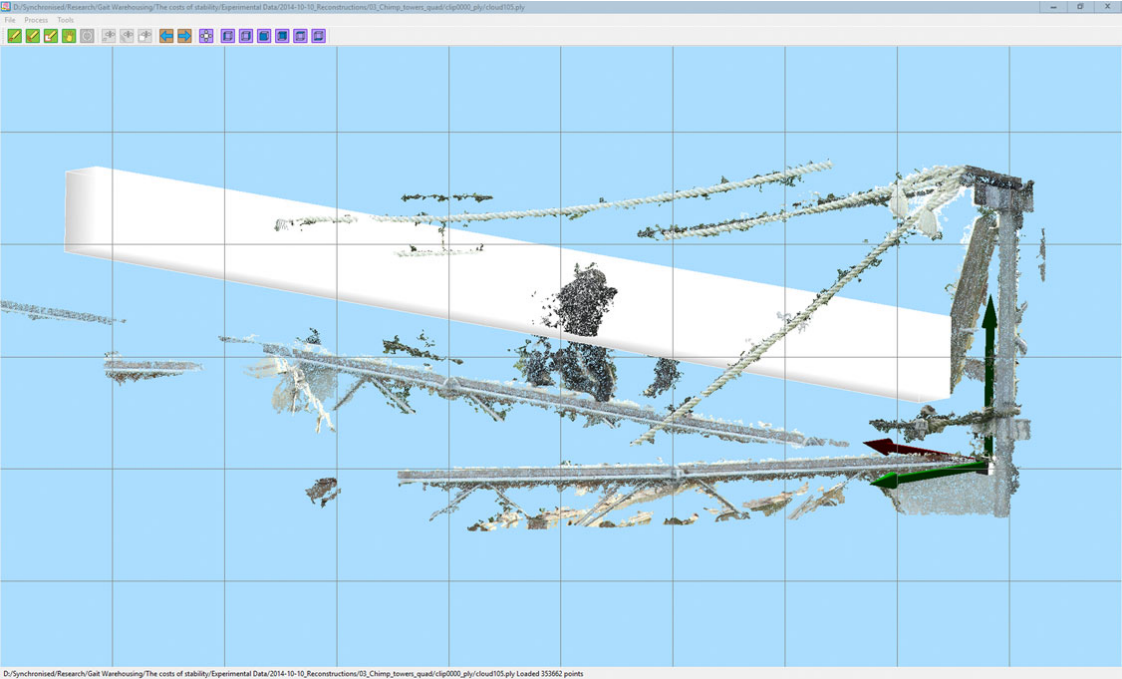
Screen shot of CloudDigitizer (www.animalsimulation.org) illustrating the point cloud reconstruction and the position of the volume of interest. The origin is set at the end of the platform with the *Z* axis oriented vertically and the *X* axis oriented in the direction of travel. The volume of interest used to measure the lateral deviation of the body point cloud is shown in white.

For verification we also needed to measure the degree of lateral movement that is actually seen in chimpanzee quadrupedal gait. We did this using our three-dimensional markerless motion capture system [43], which uses synchronized video cameras to provide frame by frame three-dimensional photogrammetric reconstructions of the free-ranging captive chimpanzees. This recording took place at the Kyoto University Primate Research Institute. The animals were walking at normal speeds on horizontal platforms and the three-dimensional photogrammetry system allows full calibration based on the measured camera separation so that no manipulation of the animals or the enclosure was required. Filming was performed using five Canon XF105 cameras filming at 720p60 synchronized using a Blackmagic Design Mini Converter Sync Generator at a distance of approximately 20 m from the outdoor climbing platform over a 2-week period in October 2014 (figure 1). The camera separation was approximately 2 m so the angular distance between each camera was approximately 5°. The raw video footage was temporally aligned (the sync generator guarantees that the individual frames align, but does not start the cameras recording at exactly the same instant) by identifying an observable action (usually a foot contact instant) in all five views. The output of this approach is calibrated and oriented point clouds representing the surface of the animal as well as the substrate. Lateral movement was calculated by defining a volume of interest using the custom written open-source software CloudDigitiser (www.animalsimulation.org) through which the flank of the animal passed while walking on the platform (figure 2). By calculating the median position of the points in the box, the forward (X) and lateral (Y) position of the flank could be automatically calculated.

## 3. Results

**Figure 3.**
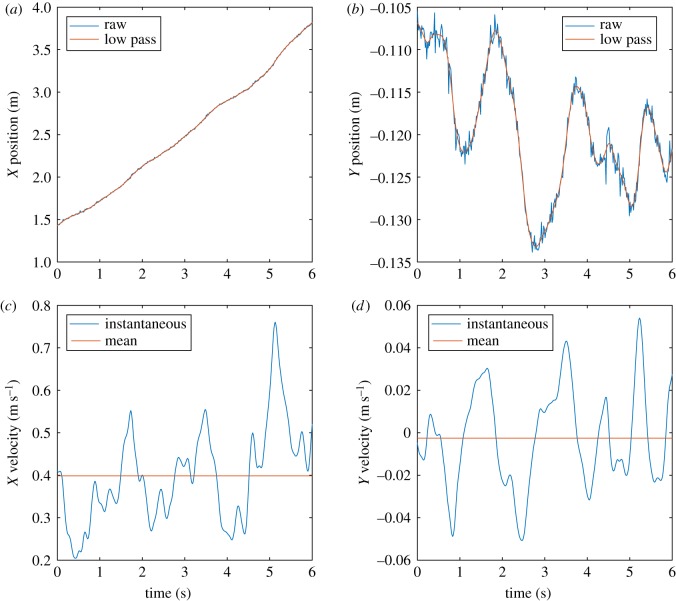
Sample positional parameters from a quadrupedal chimpanzee sequence. (*a*) Forward position; (*b*) lateral position; (*c*) forward velocity; (*d*) lateral velocity.

**Figure 4.**
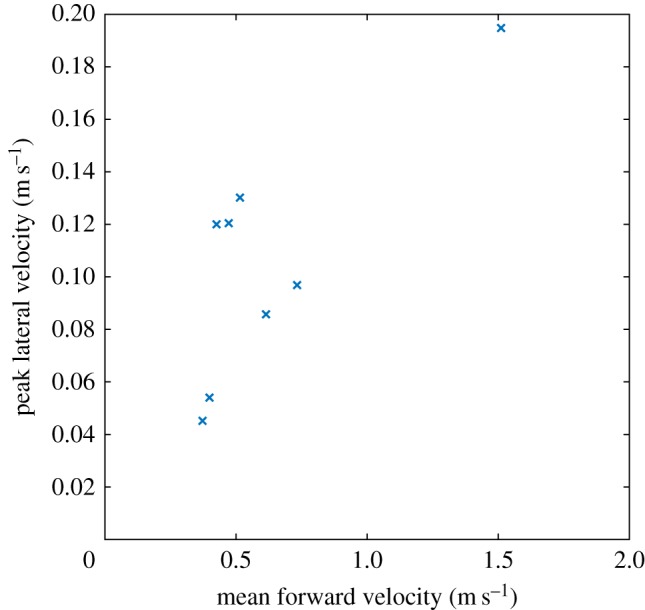
Graph showing the forward and lateral velocity characteristics of the sample quadrupedal bouts observed.

The chimpanzees in the enclosure only very rarely walked quadrupedally in the area of the enclosure where the horizontal platform was easily visible. However, we were able to obtain eight video clips where we observed steady state, straight line quadrupedal gait for three different chimpanzees. The time-dependent positions and velocities of the flank for one of the good examples are shown in figure 3. From this information, the peak lateral velocities were extracted and plotted against the mean forward velocity in figure 4 to illustrate the variation seen. The fastest quadrupedal bout has a forward velocity of 1.51 m s^−1^ and represents running. The other seven bouts are all slow, walking gaits. The peak lateral velocity varied from 0.05 to 0.19 m s^−1^, so values of 0.1 and 0.2 m s^−1^ were used as the lateral limits for the simulations.

The simulations were run as a balanced two-factor design. Factor 1 was the starting condition (diagonal or a lateral footfall sequence). Factor 2 was the amount of lateral speed restriction (no limit, less than 0.2 m s^−1^, less than 0.1 m s^−1^). For the initial case starting from a random genome, most simulations did not produce a reliable gait that could progress forward a significant distance over multiple strides, so the best five were chosen from each starting condition that showed the desired gait characteristics in terms of footfall pattern (i.e. five lateral sequence gaits and five diagonal sequence gaits were chosen). These were then further optimized using our standard procedure where each subsequent run uses a previous mid-simulation pose as its starting pose, and uses the best previous solution population as its starting solution [44]. This was repeated until no further improvement in terms of cost of locomotion was seen. Initially solutions were found for both sets of starting conditions and no lateral speed limit. The lateral limits were then applied using gait morphing where the lateral limit is incrementally applied while repeating the optimization procedure [40]. Once the required limits were obtained, the morphing process was halted and again these conditions were repeatedly optimized until no further improvement was obtained. In this way we obtained five distinct simulations for each combination of factors.

**Figure 5.**
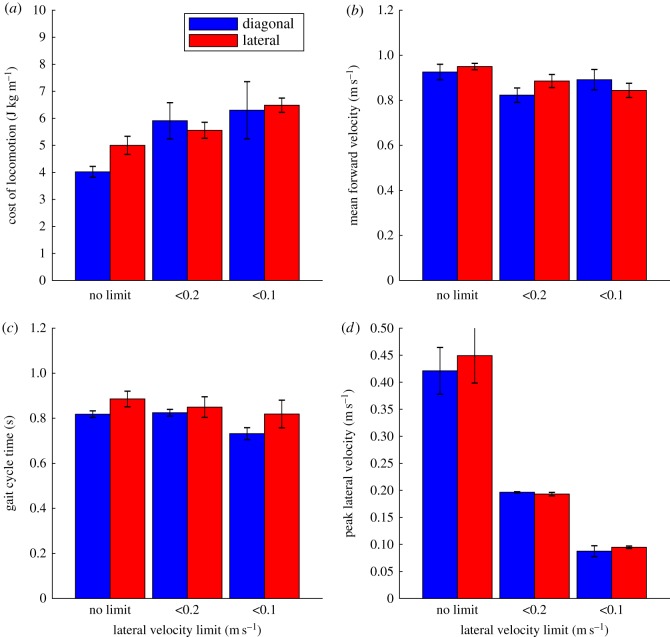
Charts showing major performance parameters for the locomotor bouts generated with different starting footfall starting conditions and under different lateral velocity limits. (*a*) Cost of locomotion; (*b*) mean forward velocity; (*c*) gait cycle time; (*d*) peak lateral velocity. The bars show the mean values of each case and the error bars represent the standard errors.

Figure 5*a* shows how the cost of locomotion is affected by the different factors. A two-way analysis of variance suggests that there is an effect of lateral velocity limit (*F* = 5.831 *p* = 0.009) but not for footfall sequence. The restriction of lateral velocity to less than 0.2 m s^−1^ causes an 18% increase in cost of locomotion and restriction to less than 0.1 m s^−1^ causes a 32% increase on average, although there is quite a high degree of variation between individual values. Figure 5*b* shows the effects on the mean forward velocity. Again, ANOVA shows a statistically significant effect of the lateral velocity limit (*F* = 4.102, *p* = 0.0293) with a 9% reduction for less than 0.2 m s^−1^ and 8% for less than 0.1 m s^−1^. This change is relatively small and may reflect the fact that the simulation's forward speed is also limited. The effects on gait cycle time are shown in figure 5*c* but no statistically significant effects were seen. Figure 5*d* shows the effects of the lateral velocity limit on the measured peak lateral velocity. As expected these limits have a major effect on the lateral velocity generated (*F* = 95.794, *p* < 0.001) but with no difference between the lateral and diagonal gaits. The peak lateral velocity seen when there is no limit is 47% of the mean forward velocity, suggesting relatively high levels of lateral movement are used in the simulation to maximize energy efficiency.

**Figure 6.**
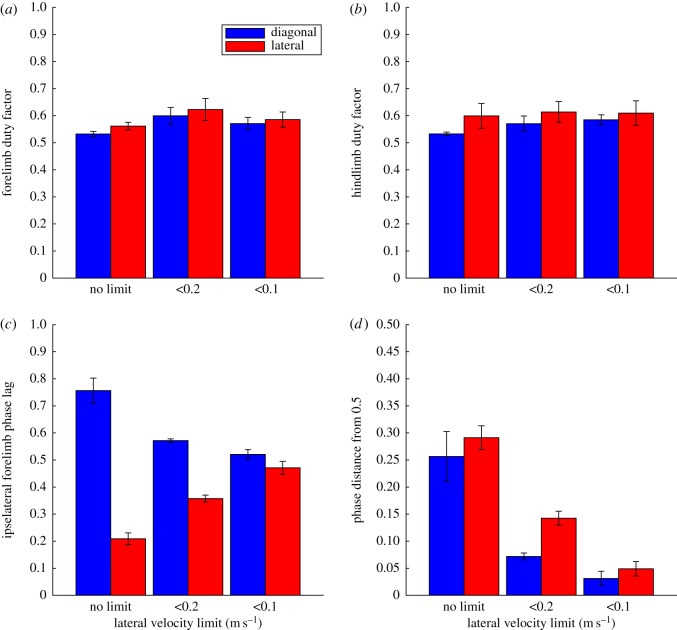
Charts showing the phase and duty factor parameters for the locomotor bouts generated with different starting footfall starting conditions and under different lateral velocity limits. (*a*) Forelimb duty factor; (*b*) hindlimb duty factor; (*c*) phase lag between hind and forelimb touchdown on the same side; (*d*) phase lag measured as the absolute difference from the trot condition. The bars show the mean values of each case and the error bars represent the standard errors.

The temporal characteristics of the generated gaits are further explored in figure 6. All the gaits generated were symmetrical so the phase differences between the left and right sides is 50%. Figure 6*a,b* show the effects on the fore and hind limb duty factors of the simulation. The only statistically significant effect here is a 13% increase in forelimb duty factor in the less than 0.2 m s^−1^ case (*F* = 3.433, *p* = 0.0488). The effects on the phase relationships between the limbs are show in figure 6*c,d*. Figure 6*c* is the phase lag of the left hand contact as a proportion (0 to 1) of the gait cycle which starts with left foot contact. Thus, diagonal gaits (left foot, right hand, right foot, left hand) have values greater than 0.5, and lateral gaits (left foot, left hand, right foot, right hand) have values less than 0.5. This is clearly seen in figure 6*c* with the expected values for diagonal and lateral gaits seen depending on the start condition (*F* = 174.858, *p* < 0.001). There is also an effect of the lateral velocity limits which moderate the effect of the starting gait condition (*F* = 50.672, *p* < 0.001 for interaction but NS as an independent effect). What appears to be happening is that the lateral velocity constraint is forcing a greater degree of diagonality, and this is further explored in figure 6*d* which shows the phase difference from 0.5 with the sign removed. This removes the effect of the starting condition almost completely (*F* = =3.935, *p* = 0.0589) and shows that the effect on both lateral and diagonal gaits in terms of increasing diagonality is largely identical (*F* = 53.059, *p* < 0.001). With the peak lateral velocity restricted to less than 0.1 m s^−1^ the difference from 0.5 is less than 5%, so this would generally be considered to be within the trotting phase space [45].

## 4. Discussion

The inclusion of a lateral stability requirement is certainly shown to improve the match between the simulated gait and the experimentally recorded gait in terms of lateral displacements. This is unsurprising because it applies an additional constraint to a model that already produces moderately convincing gait (certainly when viewed from the side). What is more interesting is the effect of this constraint on the other gait parameters in the simulated outcomes. There is a mean increase in the metabolic energy cost of locomotion of 18% for a moderate lateral stability requirement and a 32% increase for the more stringent case. The experimentally measured mean cost of locomotion for quadrupedally walking chimpanzees is 3.8 J kg^−1^ m^−1^ ranging between 2.8 and 5.8 [42] (calculated from the reported oxygen consumption using the standard conversion factor of 20.1 J ml^−1^ O_2_ [46]). The simulation's estimate is a very similar 4.81 J kg^−1^ m^−1^ with no lateral stability constraint rising to 5.74 with the less than 0.2 m s^−1^ constraint and 6.39 with the less than 0.2 m s^−1^ constraint. This may explain why previous results have led to appreciable lateral movement because it would appear that there is a real energetic cost to maintaining a laterally stable stance, and an optimization strategy that is only looking for minimal energy cost would, therefore, not identify these solutions. We would suggest that this may be due to the model taking advantage of lateral sway pendulum mechanics for additional energy recovery as has been reported in penguins [47], however without further investigation this is just speculation. In addition there are certainly sensory advantages in maintaining a platform that is stable in all three orientation axes [48], so that the choice of how much lateral movement to allow is likely to be a cost–benefit trade-off between a number of different performance goals. The somewhat smaller effect of reducing the forward velocity is rather harder to explain. The forward velocity of the model is peak limited so if the mean velocity is reduced this may reflect an increased variation in forward velocity over the gait cycle, which might be linked to the model needing to find alternative pendular systems to maximize energy recovery when the lateral pendulum is not an option. For comparison, typical walking speeds for chimpanzees are reported to vary between 0.2 and 1.2 m s^−1^ (based on tibial length calibration) [49], so the values generated by the simulation are perfectly believable. The minimal effect on cycle time would be expected because this will relate to the natural frequency of the limbs which will not be changed by the imposition of lateral velocity limits. Again for comparison, typical chimpanzee values are reported to range from 1.2 to 1.9 s [49] so these values would appear to be quite low, although within the range seen for gorillas (0.5–2.1 s [49]).

The footfall picture is equally interesting. The simulation itself is equally adept at producing both lateral and diagonal gaits and our results show very little effect of footfall sequence on any of the measured gait parameters, even though their effects on velocity and locomotor costs are substantial. Duty factors mostly fall within the experimentally observed range (0.62–0.74 [49]). Hildebrand's classic paper on primate footfall sequences [50] looked at 12 quadrupedal gait formulas for chimpanzees which ranged from 40% to 65% phase lag for the ipsilateral forefoot (generally gait is defined as: 30–45% lateral sequence, lateral couplet; 45–55% trot, 55–70% diagonal sequence, diagonal couplets [45]). More extensive work based on 50 analysed quadrupedal bouts of *Pan paniscus* produced very similar answers with phase lags in chimpanzees ranging fairly uniformly between 40% and 75% [39]. Clearly footfall sequences in chimpanzees (as well as other measures of their locomotor kinematics [51]) are much less tightly defined than seen in other primates such as Japanese macaques and spider monkeys [52], and it would be interesting to see whether our approach applied to a primate with a different body shape and locomotor habit would lead to different results. As the lateral velocity restriction is increased, both lateral sequence gaits and diagonal sequence gaits converge on a phase lag of 50% in exactly the same way. This change of the phase strongly supports the assertion that highly diagonal gaits are useful for lateral stability because they minimize the distance of the centre of mass from the support area and hence minimize the turning moments on the body that lead to excessive roll [10]. However, because chimpanzees themselves and our model are both rather agnostic about whether they use lateral or diagonal gaits, it is not possible to add a great deal to the arguments about the adaptive value of specifically diagonal gaits in primates [11,53]. However, it is entirely possible that the adaptive value is a function of arboreality and uncertainty [15] and that the current modelling paradigm would need to be altered to accommodate this.

All gait models are to some extent a simplification of reality, and the chimpanzee model presented here is no exception. Its main limitation in the context of the current work is probably the lack of mobility at the limb girdles and in the trunk itself. The latest quadrupedal robots consider trunk mobility as a key component (e.g. [54]) and the spine has long been known to be an essential part of mammal [55] and more general tetrapod [56] locomotion. Similarly, scapula shape and mobility is one of the features that distinguish hominoids from other primates [57]. It would, therefore, be very surprising if the addition of these extra structures did not have a marked effect on the simulated gaits, and the additional modelling complexity in terms of additional degrees of freedom would probably be worthwhile. Indeed, adding more flexibility might well greatly reduce the additional energetic cost of reducing the lateral sway and this would be important to quantify in future work. Unfortunately, though, while the amount of quantitative data on limb muscles is slowly being increased, the same cannot be said for axial muscles, and so a considerable amount of work would be needed for bio-realistic model construction. Another weakness of the model is that the current control strategy is open-loop. This is extremely effective in generating highly efficient gaits but the control patterns are computationally expensive to compute and not at all robust except in perfectly uniform, simulated environments. There are a number of promising quadrupedal control techniques available in the robotics literature although none are currently ideal for coping with the large numbers of muscles and degrees of freedom present in an anatomically realistic model (e.g. [58,59]). Similarly, there have been a number of recent advances of the use of neural net-based machine learning in locomotor contexts that might provide promising new methods to control bio-realistic simulations (e.g. [60,61]). An important recent advance is the understanding that limb control in animals relies on a complex interaction between neural control and mechanical specialization such that ‘mechanical intelligence' can be used to reduce the requirement for specific neural intervention [62–64]. This potentially circumvents the problems that animals have due to the low speed of neural conduction in the context of locomotion which means that tightly coupled closed-loop control is almost certainly not a practical solution for stereotyped locomotor activities. Future approaches using robotic techniques for understanding the locomotion of animals should, therefore, evaluate the payoffs of increasing the anatomical complexity of the models, as well as increasing the richness of the goal criteria (multiple objectives as well as nonlinear constraints), and also explore the different control strategies that are available to achieve these goals.

## 5. Conclusion

The realism of the gait produced by the chimpanzee model is considerably enhanced by including a lateral stability criterion and it is highly likely that this is an important evolutionary consideration. This enhanced lateral stability comes at a moderate energetic cost, however, and this cost would need to be outweighed by other adaptive advantages. The change in footfall phase relationships strongly supports arguments about the advantage of highly diagonal gaits in terms of lateral stability, but the current model does not help identify any functional advantage of diagonal over lateral gaits among primates.

## Supplementary Material

GaitSym config files for all the individual run conditions (uuencoded tgz file)

## Supplementary Material

3D outline files for the chimpanzee bones (uuencoded tgz file)

## Data accessibility

All data generated by this paper is available for free download on DataDryad along with the models used and the source code for the simulator GaitSym 2016 (http://dx.doi.org/10.5061/dryad.hq8m7 [65] and https://doi.org/10.5281/zenodo.1172654).
